# Preventive Healthcare for Women in Primary Care: A Narrative Review of Updated Screening, Risk Assessment, and Counseling Recommendations

**DOI:** 10.7759/cureus.105748

**Published:** 2026-03-24

**Authors:** Amani Altaiam, Jackleen Almomani

**Affiliations:** 1 Family Medicine, Primary Health Care Corporation, Doha, QAT

**Keywords:** breast cancer, cervical cancer screening, colon cancer prevention, hpv vaccine awareness, lung cancer, mental health services, primary care, screening program, vaccination policy, womens health

## Abstract

Women’s preventive care is a cornerstone of primary care and requires continual alignment with evolving evidence, risk stratification tools, and patient-centered counseling. This narrative review summarizes recent changes in women’s health prevention relevant to family physicians with emphasis on practical implementation in routine visits. We reviewed major recommendations and consensus guidance published between 2020 and 2025 across key domains: cancer screening (breast, lung, colorectal, cervical and selected high-risk populations), cardiovascular risk assessment and prevention, osteoporosis risk evaluation and fracture prevention, sexual and reproductive health screening and counseling, immunization in women of reproductive age and during pregnancy, mental health and substance use screening, and preventive counseling for interpersonal violence and lifestyle-related risks. Guidelines were sourced from internationally recognized authorities, including the U.S. Preventive Services Task Force (USPSTF), World Health Organization (WHO), Centers for Disease Control and Prevention (CDC), National Institute for Health and Care Excellence (NICE), and major specialty societies such as the American College of Obstetricians and Gynecologists (ACOG), American Heart Association (AHA), and International Federation of Gynecology and Obstetrics (FIGO), with preference given to the most recent updates and those directly applicable to primary care for women across the life course. Finally, we propose a pragmatic, visit-based approach for integrating updated preventive services into primary care workflows using age- and risk-based strategies and tailored counseling to improve adherence, reduce missed opportunities, and strengthen preventive care for women across the life course.

## Introduction and background

Women’s preventive health is a central component of family medicine and primary care across the life course, incorporating screening, immunization, risk assessment, and preventive counseling. Delivering high-quality prevention increasingly requires individualized decisions based on age, pregnancy status, baseline risk, comorbidities, and patient preferences, while keeping pace with evolving recommendations from major guideline bodies.

The public health relevance of women’s prevention is reflected in the burden of preventable conditions. Breast, lung, colon, and cervical cancers remain the most common cancers, supporting the need for effective screening pathways and timely follow-up [1-3]. In addition, osteoporosis and fragility fractures are major contributors to disability and reduced quality of life in women as they age, reinforcing the value of early risk identification and fracture prevention strategies in primary care [4].

Primary care is well-positioned to provide integrated preventive services through continuity, repeated patient contact, and opportunities to bundle multiple interventions into routine visits. The Women’s Preventive Services Guidelines emphasize preventive care across the life course, including services typically delivered during well-woman visits [5]. However, implementation remains challenging due to competing clinical priorities, limited visit time, frequent guideline updates, and access barriers to screening and follow-up. Several women’s preventive care recommendations have been updated in recent years, affecting primary care counseling and workflows. Women’s preventive care also includes mental health and safety. Maternal immunization is another rapidly evolving area within women’s prevention. The Centers for Disease Control and Prevention (CDC) provides guidance on vaccination during pregnancy to support the delivery of recommended vaccines and reduce missed opportunities. Newer recommendations, including maternal respiratory syncytial virus (RSV) vaccination guidance for pregnant people to protect infants from severe RSV disease, add further counseling and operational considerations in antenatal care settings [6]. Given the breadth of women’s preventive services and the pace of updates, clinicians benefit from an implementation-focused synthesis that connects recommendations to real-world primary care workflows. This narrative review summarizes updated screening, risk assessment, and counseling recommendations relevant to women’s health prevention in primary care, with emphasis on practical implementation, shared decision-making, and closing gaps in service delivery. It additionally compares and synthesizes recommendations from major international guideline bodies to highlight areas of concordance, divergence, and implications for primary care practice.

## Review

Methods

This narrative review synthesizes recommendations from major international guidelines, consensus statements, and policy updates related to women’s preventive health published between 2016 and 2026. Relevant sources were identified through targeted review of publications from internationally recognized organizations, including the U.S. Preventive Services Task Force (USPSTF), World Health Organization (WHO), Centers for Disease Control and Prevention (CDC), National Institute for Health and Care Excellence (NICE), American College of Obstetricians and Gynecologists (ACOG), American Heart Association (AHA), American Diabetes Association (ADA), International Federation of Gynecology and Obstetrics (FIGO), and other specialty societies.

Recommendations were reviewed and synthesized narratively across major preventive care domains, including cancer screening, cardiovascular risk assessment, osteoporosis prevention, reproductive and sexual health screening, mental health screening, and immunization. Priority was given to the most recent guideline updates and recommendations most applicable to primary care practice. Data were extracted manually and organized through structured comparison of guideline scope, eligibility criteria, recommended screening intervals, and implementation considerations. The synthesized findings were summarized in comparative tables to facilitate cross-guideline comparison and highlight areas of agreement and variation between international recommendations.

Review

Preventive care for women is most effectively delivered through a structured and longitudinal approach that integrates evidence-based screening, immunization, individualized risk-reduction counseling, and reliable systems to ensure follow-up of abnormal findings. The Women’s Preventive Services Initiative (WPSI) emphasizes the importance of at least one dedicated preventive care visit annually across adolescence and adulthood, with the objective of coordinating and delivering all recommended services according to a woman’s age, reproductive status, and individual risk profile [5]. In real-world primary care practice, the most effective strategy to implement these recommendations is the standardization of a comprehensive “well-woman prevention bundle.” This model incorporates pre-visit planning to identify overdue services, point-of-care shared decision-making to align care with patient values, and closed-loop follow-up systems to ensure timely evaluation of abnormal screening results.

Cancer prevention and screening

Cancer prevention constitutes a central pillar of women’s preventive health and remains a major determinant of morbidity and mortality reduction across the lifespan. Effective screening strategies are grounded in risk stratification, evidence-based intervals, and shared decision-making that aligns clinical recommendations with patient values and access considerations. A structured screening program targets breast, lung, colorectal, and cervical cancers in eligible high-risk populations, and selected hereditary cancer syndromes, each guided by evolving international consensus statements and specialty society recommendations.

For breast cancer screening, the USPSTF recommends biennial screening mammography for women aged 40-74 years at average risk, assigning a Grade B recommendation in its 2024 update [7]. This update lowered the starting age from 50 to 40 years compared with the 2016 recommendation, reflecting contemporary epidemiologic data demonstrating rising breast cancer incidence in women in their 40s and modeling evidence supporting mortality reduction with earlier initiation of screening. Current evidence is insufficient to assess the balance of benefits and harms of screening women aged 75 years or older, and similarly insufficient to support routine supplemental imaging, such as ultrasound or magnetic resonance imaging, based solely on breast density in women with otherwise normal mammograms.

The American Cancer Society (ACS) breast cancer screening guideline (2015; webpage revised 2023) adopts a graduated framework. Women aged 40-44 years may choose to begin annual screening; annual mammography is recommended for ages 45-54; and women aged ≥55 years may transition to biennial screening or continue annual screening. Screening should continue as long as a woman is in good health and has a life expectancy of at least 10 years. The ACS defines “average risk” as the absence of a personal history of breast cancer, a known genetic mutation such as BRCA1 or BRCA2, a strong family history of breast cancer, or prior chest radiation therapy before age 30 [8].

Similarly, the American College of Obstetricians and Gynecologists (ACOG) Practice Advisory updated in 2024 recommends that individuals at average risk of breast cancer should be offered screening mammography beginning at age 40 years, with screening performed every one or two years based on an informed shared decision-making process that includes discussion of the benefits and harms of annual versus biennial screening and incorporates patient values and preferences. If screening has not been initiated in the 40s, it should begin no later than age 50 years. Biennial screening, particularly after age 55 years, is considered a reasonable option to reduce potential harms, provided that patients are counseled that less frequent screening may also result in some reduction in screening benefits [9].

In contrast, the United Kingdom National Health Service (NHS) Breast Screening Programme (2025) follows a population-based model. Women are first routinely invited between ages 50 and 53 years and subsequently screened every three years. Invitations continue until age 71, after which women may self-refer for continued screening. The NHS approach emphasizes centralized organization, automatic invitations, and standardized quality assurance processes within a publicly funded health system [10].

Overall, while all major guidelines endorse mammography as the cornerstone of breast cancer screening for average-risk women, they differ in initiation age flexibility, screening frequency, and cessation criteria, reflecting variations in healthcare system structure, interpretation of benefit-harm trade-offs, and the weight placed on patient autonomy versus population-level standardization. Shared decision-making remains essential, particularly for women with prior false-positive results, limited access to screening facilities, or heightened anxiety related to cancer detection. Importantly, the effectiveness of breast cancer screening depends not only on test performance but also on the timely diagnostic evaluation of abnormal findings; therefore, structured referral pathways and tracking mechanisms are critical. Targeted prevention based on genetic risk assessment is increasingly important in cancer prevention strategies. A comparative overview of major international breast cancer screening guidelines, including those from the USPSTF, ACS, ACOG, and the United Kingdom (NHS), is summarized in Table 1.

**Table 1:** Breast cancer screening guidelines summary Abbreviations: USPSTF: U.S. Preventive Services Task Force; ACS: American Cancer Society; ACOG: American College of Obstetricians and Gynecologists; UK: United Kingdom; NHS: National Health Service (UK); BC: Breast cancer; FH: family history; BRCA: breast cancer gene (BRCA1/BRCA2); RT: radiation therapy; q2y: every two years (biennial); US: Ultrasound; MRI: Magnetic resonance imaging. Sources: Recommendations derived from guideline publications cited in references [7-10].

Guideline (Year)	Age to Start	Screening Interval
USPSTF (2024)	40 years	Biennial mammography
ACS (2015; web updated 2023)	45 years (40-44 optional)	Annual 45–54; ≥55 biennial or annual
ACOG (Practice Advisory updated 2024)	40 years	Annual or biennial
UK NHS (2025)	50 years (first invite 50-53)	Every three years

Cervical cancer screening recommendations have evolved substantially over the past decade, largely driven by advances in understanding persistent high-risk human papillomavirus (hrHPV) infection as the central etiologic factor in cervical carcinogenesis and by the increasing availability of validated HPV-based testing platforms.

In its most recent finalized recommendation (2018, reaffirmed and currently under update with a draft released in December 2024), the USPSTF recommends screening with cervical cytology (Pap smear) alone every three years for individuals aged 21-29 years. For those aged 30-65 years, three strategies are endorsed: cytology alone every three years, primary hrHPV testing every five years, or co-testing with cytology plus hrHPV testing every five years [11]. A notable proposed change in the December 2024 draft update is the inclusion of clinician-supervised self-collection of HPV samples within healthcare settings as an additional option for women aged 30-65 years. This represents a shift toward expanding access and patient acceptability, although the recommendation does not extend to unsupervised home-based testing and has not yet been finalized. Screening cessation remains recommended after age 65 in adequately screened individuals and is not advised following total hysterectomy for benign disease without prior high-grade lesions.

The ACOG most recently updated its Practice Advisory in 2021 (reaffirmed in subsequent clinical guidance), aligning largely with the USPSTF framework. ACOG continues to recommend initiating screening at age 21 and supports the three evidence-based strategies for individuals aged 30-65 years. While maintaining flexibility in implementation, ACOG emphasizes feasibility within clinical settings, shared decision-making, and adherence to established surveillance algorithms for abnormal results [12]. Some ACOG clinical materials acknowledge the expanding role of primary HPV testing, including in younger adults where validated testing platforms are available, but without formally shifting the starting age to 25 as the ACS has done.

In December 2025, the ACS issued an updated cervical cancer screening guideline that builds on its 2020 shift to primary HPV testing beginning at age 25 [13]. The 2025 update formally incorporates self-collected vaginal samples for primary high-risk HPV testing as an acceptable screening option for average-risk individuals aged 25-65 years, while maintaining clinician-collected primary HPV testing every five years as the preferred strategy when feasible. When self-collected HPV testing is used and results are negative, repeat screening is recommended at three-year intervals. The update also refines criteria for discontinuing screening after age 65 by clarifying documentation requirements for adequate prior negative testing. These changes aim to expand access, improve participation rates, and support equitable implementation of HPV-based screening strategies.

Within the United Kingdom, the NHS Cervical Screening Programme implemented a major transition beginning in 2019, moving from cytology-based screening to primary HPV testing with reflex cytology [14]. Under current programme guidance (updated through 2024-2025), individuals aged 25-64 years are invited for HPV primary screening, with routine recall every five years if HPV-negative. More frequent surveillance is undertaken for those who test HPV-positive or have abnormal cytology. Routine invitations generally cease at age 64, although individuals may continue based on clinical indication. This shift represents a system-level adoption of risk-based screening with centralized recall and quality assurance processes.

Globally, the WHO updated its cervical cancer screening guidelines in 2021 as part of its global cervical cancer elimination strategy. The WHO recommends HPV DNA testing as the preferred screening modality starting at age 30 in the general population (and earlier for women living with HIV), with screening intervals of five to ten years depending on resource settings and risk stratification [15]. The 2021 update marked a decisive move toward HPV-based approaches as the cornerstone of screening worldwide, particularly emphasizing scalable, resource-stratified implementation models for low- and middle-income countries.

Collectively, these updates illustrate a clear global transition from cytology-centered models toward HPV-based primary screening, longer intervals for HPV-negative individuals, and increasingly risk-stratified, access-oriented strategies, while differences remain in starting age thresholds, integration of self-collection, and health system delivery frameworks. A comparison of international cervical cancer screening strategies, including guidance from the USPSTF, ACS, ACOG, the UK NHS, and the WHO, is provided in Table 2.

**Table 2:** Cervical cancer screening: average-risk people with a cervix Abbreviations: USPSTF: U.S. Preventive Services Task Force; ACOG: American College of Obstetricians and Gynecologists; ACS: American Cancer Society; NHS: National Health Service; WHO: World Health Organization; HPV: Human papillomavirus; hrHPV: high-risk human papillomavirus; CIN2+: cervical intraepithelial neoplasia grade 2 or higher. Sources: Recommendations derived from guideline publications cited in references [11-15].

Guideline (Year)	Starting Age	Preferred Strategy	Acceptable Alternatives	Stop Age	Key Notes
USPSTF (2018; draft update 2024)	21 years	21-29: Cytology every three years	30-65: Primary hrHPV every five years, co-testing every five years, or cytology every three years	>65 if adequately screened	Draft 2024 includes clinician-supervised self-collection (not yet finalized). No screening after total hysterectomy for benign disease and no history of CIN2+ [11]
ACOG (2021, reaffirmed)	21 years	Aligns with USPSTF framework	Supports primary HPV testing where validated platforms are available	>65 if adequately screened	Emphasizes feasibility, shared decision-making, and adherence to management algorithms [12]
ACS (2025)	25 years	Primary HPV testing every five years	If self-collected HPV (healthcare setting): repeat every three years if negative; if HPV unavailable: co-testing every five years or cytology every three years	>65 if adequately screened	Formal incorporation of the self-collection pathway; clarified documentation criteria for discontinuation [13]
UK NHS Cervical Screening Programme (2025)	25 years	Primary HPV testing with reflex cytology	Routine recall every five years if HPV-negative	Routine invitations cease at 64	Centralized recall system; risk-based surveillance for HPV-positive results [14]
WHO (2021)	30 years	HPV DNA testing preferred	Resource-stratified options where HPV testing is unavailable	Country-specific	Emphasizes HPV-based screening as the cornerstone of the global cervical cancer elimination strategy. [15]

For colorectal cancer screening, the USPSTF in its May 2021 update recommends routine colorectal cancer (CRC) screening for adults aged 50-75 years (Grade A) and screening for adults aged 45-49 years (Grade B). Screening for adults aged 76-85 years should be individualized based on overall health, prior screening history, and patient preferences. Compared with the prior USPSTF 2016 recommendation, the key update was lowering the starting age from 50 to 45, driven by rising CRC incidence in younger adults, while continuing an individualized approach after age 75.

The ACS guideline released in 2018 recommends that average-risk adults begin regular CRC screening at age 45 using either high-sensitivity stool-based tests or structural examinations depending on patient preference and access. Screening should continue through age 75, with individualized decisions for ages 76-85 and discontinuation after age 85. Compared with previous ACS guidance, the major change in 2018 was lowering the starting age from 50 to 45 and emphasizing that the most effective test is the one that patients are willing to complete, with colonoscopy required for any positive non-colonoscopy test.

The UK NHS Bowel Cancer Screening Programme uses a mailed fecal immunochemical test (FIT) home kit within an organized national recall system. Current programme guidance states that individuals aged 50-74 years are invited every two years, and those aged ≥75 years may request screening kits. Compared with the earlier programme that primarily invited individuals aged 60-74 years, England has gradually lowered the starting age from 60 to 50, with rollout beginning in 2021 and entering its final phase in 2025. A January 2026 implementation update also describes lowering the FIT threshold (e.g., from 120 to 80 μg Hb/g) to increase screening sensitivity, with phased rollout planned through 2028.

The American College of Gastroenterology (ACG) updated its colorectal cancer screening guideline in 2021, recommending that average-risk adults begin screening at age 45 years and continue through age 75. Screening decisions for individuals aged 76-85 should be individualized according to health status and prior screening history. The ACG recommends colonoscopy every 10 years and annual fecal immunochemical testing as preferred primary screening strategies, while also recognizing multitarget stool DNA testing every three years, CT colonography every five years, flexible sigmoidoscopy every five to ten years, and colon capsule endoscopy every five years as alternative screening options.

A comparison of colorectal cancer screening recommendations from the USPSTF, ACS, and the UK NHS and ACG bowel screening guidelines is presented in Table 3.

**Table 3:** Colorectal cancer screening: average-risk adults Abbreviations: USPSTF: U.S. Preventive Services Task Force; ACS: American Cancer Society; NHS: National Health Service; ACG: American College of Gastroenterology; FIT: fecal immunochemical test; gFOBT: guaiac fecal occult blood test; CT: computed tomography. Sources: Recommendations derived from guideline publications cited in references [16-19].

Guideline (Year)	Age to Start	Recommended Modalities and Intervals	Age to Stop / Modify	Key Implementation Notes
USPSTF (2021)	45 years	Annual fecal immunochemical test (FIT) or high-sensitivity guaiac fecal occult blood test (gFOBT); stool DNA–FIT every 1-3 years; colonoscopy every 10 years; CT colonography every five years; flexible sigmoidoscopy every five years (or every 10 years + annual FIT)	Routine to 75; 76-85 individualized	Any abnormal non-colonoscopy test requires a diagnostic colonoscopy [16]
ACS (2018)	45 years	Colonoscopy every 10 years; CT colonography every five years; flexible sigmoidoscopy every five years; stool-based tests supported	Continue through 75; 76-85 individualized; >85 stop	Emphasizes adherence to any recommended test [17]
UK NHS Bowel Cancer Screening Programme (2021–2026 rollout)	50 years	Mailed home FIT every two years	Invited to 74; ≥75 may request	Organized population programme; phased lowering of start age from 60 to 50; recent FIT sensitivity adjustment to increase early detection [18]
ACG (2021)	45 years	Preferred: colonoscopy every 10 years or annual FIT; alternatives include stool DNA every 3 years, CT colonography every 5 years, flexible sigmoidoscopy every 5–10 years, or colon capsule every 5 years	Strongly recommended 50-75; 45-49 suggested; >75 individualized	Prioritizes colonoscopy and FIT as first-line; positive stool tests require colonoscopy [19]

Lung cancer is the leading cause of cancer-related mortality among women worldwide, and screening is therefore targeted toward individuals at sufficiently elevated risk to justify the balance of benefits and harms [20]. The USPSTF updated its recommendation in 2021 to advise annual low-dose computed tomography (LDCT) for adults aged 50-80 years with a ≥20 pack-year smoking history who currently smoke or have quit within the past 15 years (Grade B recommendation). Screening should be discontinued once a person has not smoked for 15 years or develops a health condition that substantially limits life expectancy or the ability to undergo curative lung surgery. This update lowered both the starting age (from 55 to 50 years) and pack-year threshold (from 30 to 20 pack-years) compared with the 2013 recommendation, aiming to improve early detection and reduce disparities, particularly among women and racial minorities [21].

The ACS updated its guideline in 2023 and now recommends annual LDCT for adults aged 50-80 years with a ≥20 pack-year history who currently smoke or formerly smoked, without imposing a strict 15-year cessation cutoff. The ACS removed the quit-time limit to expand eligibility for individuals who remain at elevated risk despite longer periods of abstinence, while continuing to emphasize shared decision-making and integration of structured smoking cessation support [22].

The Canadian Task Force on Preventive Health Care (CTFPHC) recommends annual LDCT for adults aged 55-74 years with a ≥30 pack-year history who currently smoke or quit within the past 15 years (2016 guideline, reaffirmed with contextual updates pending). The Canadian approach includes strong emphasis on programmatic screening delivered within organized systems with radiologic quality assurance and standardized reporting (e.g., Lung-RADS-type frameworks), alongside mandatory smoking cessation interventions [23].

In the United Kingdom, lung cancer screening has transitioned from pilot programs to broader implementation through the NHS England Targeted Lung Health Check Programme, which offers LDCT to adults typically aged 55-74 years with a current or former smoking history, identified through primary care records and risk assessment tools (such as PLCOm2012 risk modeling). Unlike purely age/pack-year thresholds, the UK model incorporates individualized risk prediction algorithms to improve cost-effectiveness and reduce unnecessary imaging [24].

Across all major guidelines, LDCT remains the only modality with demonstrated mortality reduction in high-risk populations, supported by large randomized trials such as the National Lung Screening Trial (NLST) and the NELSON ((NEderlands-Leuvens Longkanker Screenings ONderzoek) trial. However, screening carries potential harms, including false-positive results, incidental findings, radiation exposure, and overdiagnosis. Accordingly, eligibility assessment, structured shared decision-making, smoking cessation counseling, and clearly defined referral pathways for abnormal findings are essential components of safe and effective implementation. Table 4 summarizes the comparative eligibility criteria and system-level differences among major international recommendations.

**Table 4:** Lung cancer screening recommendations Abbreviations: USPSTF: U.S. Preventive Services Task Force; ACS: American Cancer Society; CTFPHC: Canadian Task Force on Preventive Health Care; NHS: National Health Service; LDCT: low-dose computed tomography; PLCO: prostate, lung, colorectal, and ovarian Sources: Recommendations derived from guideline publications cited in references [21-24].

Guideline (Year)	Eligible Age	Smoking History	Modality & Interval	When to Stop	Key Implementation Features
USPSTF (2021)	50-80 years	≥20 pack-years; current smoker or quit within 15 years	Annual low-dose computed tomography (LDCT)	Stop if >15 years since quitting or if health limits curative surgery	Lowered age (55→50) and pack-years (30→20) from the 2013 recommendation; emphasizes shared decision-making and smoking cessation counseling [21]
ACS (2023)	50-80 years	≥20 pack-years; current or former smoker (no strict 15-year quit limit)	Annual LDCT	Discontinue if serious comorbidities limit life expectancy or ability for curative treatment	Removed 15-year cessation cutoff; prioritizes broader eligibility and integrates smoking cessation interventions [22]
CTFPHC (2016)	55-74 years	≥30 pack-years; current smoker or quit within 15 years	Annual LDCT (often within structured screening programs)	Stop after age 74 or if health status precludes benefit	Recommends screening within organized programs with radiologic quality standards and cessation support [23]
NHS England Targeted Lung Health Check Programme (2024–2025 rollout)	55-74 years	Current or former smokers identified through primary care records and risk prediction tools (e.g., PLCOm2012 model)	Risk-stratified LDCT screening	Based on individualized risk assessment and clinical fitness	Uses centralized invitations and validated risk calculators rather than strict pack-year thresholds alone [24]

Bone health and fracture prevention

Recent international osteoporosis screening guidelines emphasize risk-based assessment and targeted screening, with updates published between 2021 and 2025. The USPSTF released its latest recommendation in 2025, advising routine screening for osteoporosis in women aged ≥65 years and in postmenopausal women younger than 65 years who have increased fracture risk, typically assessed using clinical risk tools followed by dual-energy X-ray absorptiometry (DXA) testing [25]. In Canada, the Canadian Task Force on Preventive Health Care (2023) recommends a risk-assessment-first approach, particularly for women aged ≥65 years, while generally advising against routine screening in women aged 40-64 years and men aged ≥40 years without significant risk factors because the potential harms may outweigh benefits [26].

European guidance similarly prioritizes risk-stratified approaches. The National Osteoporosis Guideline Group (NOGG) in the United Kingdom, updated in 2024, recommends the use of the FRAX (Fracture Risk Assessment Tool) to estimate the 10-year probability of major osteoporotic fractures and determine which individuals require DXA scanning and/or pharmacologic therapy, particularly among postmenopausal women and men aged ≥50 years [27]. In Australia, the Royal Australian College of General Practitioners (RACGP) and Healthy Bones Australia guideline (updated 2024 and published in 2025) advises against universal population-based screening and instead promotes case-finding with fragile X syndrome (FRAX)-based risk stratification to guide further testing [28]. In the United States, women’s health guidance from ACOG, last updated in 2021, provides clinical recommendations on osteoporosis prevention, screening, and diagnosis in postmenopausal patients, including the use of bone mineral density testing and preventive lifestyle strategies [29].

Regionally, the Qatar national guideline for fracture risk assessment and management also adopts a FRAX-based strategy, recommending targeted risk assessment among postmenopausal women and men older than 50 years and discouraging routine DXA screening in individuals without clinical risk factors [30]. Overall, despite regional differences, most recent guidelines converge on three major principles: targeted screening rather than universal population screening, the use of fracture-risk prediction tools such as FRAX, and prioritization of screening in postmenopausal women and older adults at elevated fracture risk. A comparative summary of major osteoporosis screening guidelines from North America, Europe, Australia, and the Middle East is provided in Table 5.

**Table 5:** Osteoporosis screening guidelines Abbreviations: USPSTF: U.S. Preventive Services Task Force; NOGG: National Osteoporosis Guideline Group; RACGP: Royal Australian College of General Practitioners; ACOG: American College of Obstetricians and Gynecologists; FRAX: Fracture Risk Assessment Tool; DXA: Dual-Energy X-ray Absorptiometry. Sources: Recommendations derived from guideline publications cited in references [25-30].

Guideline (Year)	Target Population	Key Screening Recommendation
USPSTF (2025)	Women ≥65 years; postmenopausal women <65 with increased fracture risk	Recommends osteoporosis screening using bone mineral density testing to prevent fractures. Evidence is insufficient to assess benefits and harms of screening in men [25]
Canadian Task Force on Preventive Health Care (2023)	Community-dwelling adults ≥40 years	Recommends a risk-assessment–first approach in women ≥65 years and recommends against routine screening in women 40-64 years and men ≥40 years without significant risk factors [26]
NOGG (2024)	Postmenopausal women and men ≥50 years	Recommends FRAX-based fracture risk assessment to determine the need for DXA scanning and pharmacologic treatment [27]
RACGP and Healthy Bones Australia (2024/2025)	Postmenopausal women and men >50 years	Does not recommend population-based screening; instead promotes clinical case-finding and FRAX-based risk stratification to guide DXA testing [28]
ACOG Clinical Practice Guideline (2021)	Postmenopausal women	Provides guidance on screening, diagnosis, and prevention of osteoporosis, including bone mineral density testing and preventive strategies [29]
Qatar National Guideline (2024)	Postmenopausal women and men >50 years	Recommends FRAX-based fracture risk assessment using the Qatar-specific FRAX model and discourages routine DXA screening without clinical risk factors [30]

Chronic disease prevention

Chronic diseases remain a leading contributor to morbidity and mortality among women worldwide and represent a major focus of preventive care in primary care settings. Conditions such as hypertension, diabetes mellitus, and atherosclerotic cardiovascular disease (ASCVD) account for a substantial proportion of preventable illness and death. Early identification and risk modification through screening, lifestyle interventions, and evidence-based pharmacologic therapy are central components of effective prevention strategies.

Major organizations, including the USPSTF, American College of Cardiology (ACC), American Heart Association (AHA), and American Diabetes Association (ADA), have issued updated recommendations that guide clinicians in implementing these preventive measures within routine clinical practice. Hypertension screening is a cornerstone of chronic disease prevention. The USPSTF recommends routine office-based blood pressure screening, with confirmation of elevated readings using out-of-office measurements, such as home or ambulatory blood pressure monitoring, before initiating treatment [31]. Similarly, ACC/AHA hypertension guidelines emphasize the importance of self-measured and ambulatory blood pressure monitoring both for confirming the diagnosis and for guiding ongoing management [32].

In parallel, screening for diabetes mellitus is also emphasized to identify individuals at risk of diabetes and related complications. The USPSTF recommends screening for prediabetes and type 2 diabetes in adults aged 35-70 years with overweight or obesity, followed by referral to effective preventive interventions for individuals with prediabetes [33]. Complementing this approach, the ADA Standards of Care recommend that screening begin no later than age 35 for all adults, with earlier testing in individuals who are overweight or obese and have additional risk factors. When results are normal, repeat screening is typically performed at approximately three-year intervals, although shorter intervals may be appropriate for higher-risk individuals [34]. For individuals identified with prediabetes, prevention strategies focus primarily on lifestyle modification, including structured programs aimed at weight reduction and increased physical activity. The ADA further recommends considering metformin therapy for diabetes prevention in higher-risk individuals, particularly adults aged 25-59 years with a body mass index (BMI) ≥35 kg/m², higher fasting glucose or glycated hemoglobin (A1C), and those with a history of gestational diabetes [35]. Cardiovascular risk reduction is another critical component of chronic disease prevention.

The USPSTF recommends statin therapy for primary prevention of ASCVD in adults aged 40-75 years who have at least one cardiovascular risk factor and an estimated 10-year ASCVD risk of ≥10%, while selectively offering statins to individuals with a 10-year risk of 7.5% to <10% [36]. The ACC/AHA cholesterol management guideline similarly emphasizes formal ASCVD risk assessment and clinician-patient shared decision-making, with statins serving as the first-line pharmacologic therapy in appropriately selected individuals [37]. Finally, the role of aspirin in primary prevention has become more limited and requires careful individualization. Consistent with the USPSTF recommendations, the ACC/AHA guideline advises that aspirin should be used infrequently for routine primary prevention, may be considered selectively in certain higher-risk adults without increased bleeding risk, and should generally be avoided in older adults or those with elevated bleeding risk [38,39]. Collectively, these recommendations highlight the importance of risk-based screening, patient-centered counseling, and targeted preventive interventions in reducing the burden of chronic diseases among women across the life course.

Reproductive and sexual health preventive services

Reproductive and Sexual Health Preventive Services are a fundamental component of women’s health care across the life course. These services aim to promote healthy pregnancies, reduce maternal and neonatal complications, prevent sexually transmitted infections (STIs), and support mental health during the perinatal period. Major organizations such as the USPSTF, ACOG, WHO, and CDC provide evidence-based guidance for integrating these services into routine clinical care.

Within primary care and obstetric practice, preventive strategies focus on screening for pregnancy-related complications, infection prevention, and early identification of psychosocial risks, thereby improving both short- and long-term maternal and child health outcomes. Preventive care during pregnancy and the postpartum period is particularly important because timely interventions can substantially reduce adverse outcomes. The USPSTF recommends one-time screening for gestational diabetes mellitus (GDM) at or after 24 weeks of gestation, concluding that current evidence is insufficient to support routine screening earlier in asymptomatic pregnant individuals [40]. Early identification of GDM allows clinicians to implement dietary management, glucose monitoring, and pharmacologic therapy when needed, thereby reducing the risk of fetal macrosomia, birth trauma, and maternal complications. Another key preventive intervention involves the reduction of preeclampsia risk. For pregnant individuals who are at increased risk, the USPSTF and ACOG recommend low-dose aspirin (81 mg daily) beginning after 12 weeks of gestation, which has been shown to decrease the incidence of preeclampsia, preterm birth, and related maternal morbidity [41][42]. Mental health prevention is also increasingly recognized as an essential component of perinatal care. The USPSTF recommends counseling interventions for pregnant and postpartum individuals at increased risk of perinatal depression, particularly structured programs based on cognitive behavioral therapy or interpersonal therapy, which have demonstrated effectiveness in reducing the likelihood of depressive episodes during the perinatal period [43].

Screening for infectious diseases represents another cornerstone of reproductive and sexual health prevention. The USPSTF recommends routine screening for chlamydia and gonorrhea in all sexually active women aged 24 years or younger, as well as in older women with increased risk factors such as new or multiple sexual partners. Early detection and treatment of these infections are crucial to prevent complications, including pelvic inflammatory disease, infertility, and adverse pregnancy outcomes. International guidance from the WHO similarly emphasizes targeted screening strategies, particularly in populations with higher infection prevalence or limited access to health services. Screening for human immunodeficiency virus (HIV) is also recommended as part of routine preventive care.

The USPSTF and CDC advise at least one lifetime HIV screening test for adolescents and adults aged 15 to 65 years, with repeat testing for individuals at increased risk and universal screening during pregnancy when HIV status is unknown, enabling early treatment and prevention of vertical transmission. Screening recommendations for hepatitis C virus (HCV) infection have expanded considerably in recent years in response to the availability of highly effective antiviral therapies and global elimination initiatives. This approach aligns closely with recommendations from the CDC and WHO, which emphasize broad population screening to facilitate early diagnosis and linkage to curative treatment. Collectively, these preventive strategies highlight the importance of integrating reproductive and sexual health screening into routine primary care visits rather than limiting testing to symptomatic presentations or isolated risk-based encounters. A comparison of international infection screening strategies from the USPSTF, CDC, the United Kingdom, and the WHO is summarized in Table 6.

**Table 6:** Infection screening recommendations for women Abbreviations: USPSTF: U.S. Preventive Services Task Force; CDC: Centers for Disease Control and Prevention; NHS: National Health Service; WHO: World Health Organization; HBV: hepatitis B virus; HCV: hepatitis C virus; HIV: human immunodeficiency virus; NCSP: National Chlamydia Screening Programme; STI: sexually transmitted infection. Sources: Recommendations derived from guideline publications cited in references [44–58].

Guideline Organization	Chlamydia and Gonorrhea	HIV	Hepatitis C	Pregnancy Infectious Disease Screening (HIV, HBV, Syphilis)
USPSTF	Screen sexually active women ≤24 years and women ≥25 years with increased risk; the recommendation also applies to pregnant persons. Evidence remains insufficient to recommend routine screening in men [44].	Screen adolescents and adults aged 15–65 years; screen younger or older individuals if increased risk is present. All pregnant persons with unknown HIV status should also be screened, including those presenting during labor [45].	One-time screening recommended for adults aged 18–79 years, including pregnant individuals; repeat testing based on ongoing risk factors. [46].	Addressed across multiple statements: universal hepatitis B screening at the first prenatal visit, universal early syphilis screening during pregnancy, and HIV screening during pregnancy. [45,47,48].
CDC	Provides detailed STI screening guidance based on population risk and clinical settings, including pregnancy-specific recommendations such as screening early in pregnancy and repeat testing in higher-risk individuals [49].	Recommends that everyone aged 13-64 years be tested for HIV at least once as part of routine health care, with more frequent testing for individuals with ongoing risk; opt-out testing approaches are encouraged [49].	Recommends universal hepatitis C screening for all adults ≥18 years and screening during each pregnancy, with repeat testing for individuals with continuing risk factors [49].	Provides infection-specific pregnancy screening guidance, including syphilis screening at the first prenatal visit and other infection-specific recommendations within STI treatment guidelines [49].
United Kingdom (NHS / UK Programs)	The National Chlamydia Screening Programme (NCSP) promotes opportunistic testing among individuals <25 years, combined with partner notification and retesting after treatment [50].	Antenatal screening programs include HIV testing (along with hepatitis B and syphilis) early in pregnancy, with opportunities to repeat or re-offer testing later in pregnancy if initially declined [51].	UK guidance primarily supports risk-based hepatitis C testing (e.g., individuals with previous blood transfusions, injecting drug use, or origin from higher-prevalence regions) according to NHS and NICE guidance [52].	NHS antenatal screening offers routine blood tests for HIV, hepatitis B, and syphilis in every pregnancy, ideally early in pregnancy, with systems to re-offer testing if initially declined. [53].
WHO	WHO guidance supports screening and management strategies for Chlamydia trachomatis and Neisseria gonorrhoeae, emphasizing adaptation of screening programs according to epidemiologic context and available resources [54].	WHO promotes expanded access to HIV testing through integrated services, community testing models, and HIV self-testing, with linkage to prevention and treatment services [55].	WHO recommends expanded hepatitis C testing strategies to support elimination goals, including simplified diagnostic pathways and reflex viral load testing to improve linkage to treatment [56].	WHO “triple elimination” initiatives emphasize antenatal testing for HIV, syphilis, and hepatitis B combined with treatment and prevention strategies to reduce mother-to-child transmission [57,58].

Mental health preventive services

Mental health and personal safety are integral components of comprehensive preventive care and play a critical role in overall well-being, functional status, and quality of life. Mental health disorders such as depression and anxiety are among the leading causes of disability worldwide and are associated with significant social, physical, and economic consequences if left unrecognized or untreated. Preventive screening in primary care settings allows for early identification of individuals at risk and facilitates timely intervention, referral, and ongoing support. Women may be particularly vulnerable during periods of life transition, including pregnancy and the postpartum period, when hormonal, psychosocial, and environmental factors can increase susceptibility to mood and anxiety disorders. As a result, preventive care frameworks increasingly emphasize the integration of mental health assessment into routine health visits, including prenatal and postpartum care.

The USPSTF recommends routine screening for depression in all adults, including pregnant and postpartum individuals, provided that adequate systems are in place to ensure accurate diagnosis, effective treatment, and appropriate follow-up. Common screening tools used in clinical practice include validated instruments such as the Patient Health Questionnaire (PHQ-9) and the Edinburgh Postnatal Depression Scale (EPDS) for perinatal populations. Early identification of depressive symptoms enables clinicians to initiate interventions such as psychotherapy, pharmacotherapy, or referral to mental health services, thereby reducing the risk of chronic depression and associated functional impairment. Although suicide represents a major public health concern and is a leading cause of death globally, the USPSTF currently concludes that the evidence is insufficient to recommend universal suicide risk screening in all adults, highlighting the need for further research on effective screening strategies and downstream interventions [59].

Screening for anxiety disorders has also gained increasing recognition as part of preventive mental health care. The USPSTF supports screening for anxiety disorders in adults, including pregnant and postpartum individuals, based on evidence demonstrating a moderate net benefit from early detection and treatment [60]. Anxiety disorders often coexist with depression and can significantly affect daily functioning, maternal-infant bonding, and adherence to medical care during pregnancy and the postpartum period. However, evidence supporting routine screening among older adults remains limited, and further research is needed to clarify optimal screening approaches in this population.

Personal safety and protection from violence are equally important aspects of preventive care. Intimate partner violence (IPV) is a major public health issue that affects millions of women worldwide and is associated with numerous adverse health outcomes, including physical injury, mental health disorders, substance misuse, and poor reproductive outcomes. The USPSTF recommends screening for intimate partner violence in women of reproductive age, including those who are pregnant or postpartum, and providing or referring individuals who screen positive to ongoing support services and evidence-based interventions [61]. Effective implementation of IPV screening requires trauma-informed clinical practices, confidential environments that support safe disclosure, and clearly defined referral pathways to social services, counseling, and community resources. Integrating mental health screening and personal safety assessments into routine preventive visits allows clinicians to address both psychological well-being and social determinants of health, thereby promoting a more comprehensive and patient-centered approach to preventive care.

Immunizations

Immunization is a cornerstone of preventive health care for women across the life course and plays a particularly important role during pregnancy, when vaccination protects both the mother and the infant through transplacental antibody transfer. Major public health organizations, including the Centers for Disease Control and Prevention, the Advisory Committee on Immunization Practices, and the World Health Organization, recommend a series of age- and risk-based vaccines for adult women, as well as specific vaccines during pregnancy to prevent severe maternal illness and neonatal complications. Vaccination during pregnancy has been shown to reduce the risk of maternal hospitalization and to provide passive immunity to the newborn during the early months of life when infants are most vulnerable to infectious diseases.

The CDC recommends that all pregnant individuals receive the inactivated influenza vaccine during any trimester of pregnancy to reduce the risk of severe influenza infection and associated complications such as hospitalization and preterm birth [62]. Seasonal influenza vaccination has been demonstrated to decrease both maternal illness and influenza infection in infants during the first months of life. In addition, tetanus-diphtheria-acellular pertussis (Tdap) vaccination is recommended during each pregnancy, ideally between 27 and 36 weeks of gestation, to maximize maternal antibody response and transplacental transfer to the fetus, thereby protecting newborns from pertussis in early infancy [63].

More recently, vaccination recommendations have expanded to include COVID-19 vaccination for pregnant individuals, which is recommended by the CDC and ACIP because pregnancy increases the risk of severe COVID-19 illness and adverse pregnancy outcomes. Vaccination during pregnancy has been shown to reduce maternal morbidity and provide passive antibody protection to the infant [64]. In addition, the maternal RSV vaccine has been recommended in certain countries for administration during 32-36 weeks of gestation to reduce the risk of severe RSV disease in infants during the first months of life [65].

Beyond pregnancy, routine adult immunization remains essential for women throughout adulthood. Vaccination against HPV is recommended for adolescents and young adults to prevent cervical cancer and other HPV-associated malignancies. Additional recommended vaccines include measles-mumps-rubella (MMR) and varicella for individuals without evidence of immunity, hepatitis A and B vaccines for individuals with risk factors, and age-based vaccines such as herpes zoster and pneumococcal vaccines in older adults. These vaccines help prevent infections that can cause substantial morbidity, especially among older individuals or those with underlying health conditions [66]. Integrating vaccination into routine preventive visits ensures that women receive appropriate immunizations according to age, risk factors, and reproductive status. A comprehensive summary of recommended vaccines for adult females, including pregnant individuals, is presented in Table 7.

**Table 7:** Recommended vaccines for adult females including pregnant women. COVID-19: Coronavirus disease 2019; CDC: Centers for Disease Control and Prevention; Tdap: Tetanus toxoid, reduced diphtheria toxoid, and acellular pertussis vaccine; RSV: Respiratory syncytial virus; HPV: Human papillomavirus; MMR: Measles, mumps, and rubella; ACIP: Advisory Committee on Immunization Practices; RZV: Recombinant zoster vaccine.

The Vaccine Name	Target Group	Key Recommendation
Influenza (inactivated) vaccine	All pregnant individuals and adults annually	Recommended during any trimester of pregnancy and annually for all adults.
Tdap vaccine	Pregnant individuals	One dose during each pregnancy, ideally at 27-36 weeks of gestation.
COVID-19 vaccine	Pregnant individuals and all adults	Recommended according to the current CDC/ACIP schedule and risk profile.
Maternal RSV vaccine	Pregnant individuals	Recommended at 32-36 weeks of gestation in settings where available.
HPV vaccine	Adolescents and adults ≤26 years (and some adults aged 27-45 years)	Prevents cervical and other HPV-related cancers; not given during pregnancy.
MMR vaccine	Adults without evidence of immunity	Contraindicated during pregnancy; recommended pre-pregnancy if nonimmune.
Varicella vaccine	Adults without immunity	Administer before pregnancy; contraindicated during pregnancy.
Hepatitis A vaccine	Adults at risk (travel, chronic liver disease, exposure risk)	Risk-based vaccination.
Hepatitis B vaccine	Adults without prior vaccination or with risk factors	Universal adult vaccination is recommended.
Zoster (shingles) vaccine	Adults ≥50 years	Recombinant zoster vaccine recommended.
Pneumococcal vaccine	Older adults or high-risk individuals	Risk- and age-based recommendations.

In summary, preventive services for women encompass a broad set of evidence-based strategies aimed at promoting health, preventing disease, and enabling early detection across the life course. These services include screening for chronic diseases, reproductive and sexual health conditions, infectious diseases, mental health disorders, and interpersonal safety concerns, as well as age- and risk-appropriate immunization. When implemented systematically through structured preventive visits in primary care, these recommendations facilitate early identification of risk factors, timely intervention, and improved outcomes for women during adolescence, reproductive years, pregnancy, and older adulthood. Effective implementation also requires patient-centered counseling, shared decision-making, and access to multidisciplinary support services. Together, these integrated preventive strategies form the foundation of comprehensive women’s health care and support long-term well-being across the lifespan.

Limitations

This review has several limitations. It focuses on major international guidelines and consensus recommendations relevant to women’s preventive care in primary care settings; therefore, some regional or specialty-specific guidance may not have been included. In addition, preventive recommendations may vary across countries due to differences in healthcare systems, population risk profiles, and resource availability. As a result, implementation of these strategies may require adaptation to local clinical practice and health system context.

## Conclusions

Women’s preventive health in primary care is most effective when delivered as a coordinated, life-course package that combines evidence-based screening, immunization, risk assessment, and targeted counseling while accounting for individual risk factors, pregnancy status, comorbidities, and patient preferences. As recommendations continue to evolve, clinicians can reduce missed opportunities by using standardized workflows such as pre-visit planning, clear eligibility algorithms, standing orders for high-value services, and reliable tracking systems for abnormal results and follow-up. Integrating shared decision-making, particularly when guidelines differ or when benefits and harms are closely balanced, supports patient-centered choices and improves adherence. Ultimately, a practical, systems-based approach that aligns guideline updates with clinic processes can strengthen prevention across the female lifespan, improve equity in access and uptake, and translate recommendations into measurable health gains.
